# Stretched Radial Trajectory Design for Efficient MRI with Enhanced K-Space Coverage and Image Resolution †

**DOI:** 10.3390/bioengineering12111152

**Published:** 2025-10-24

**Authors:** Li Song Gong, Zihan Zhou, Qing Li, Yurui Qian, Yang Yang, Kawin Setsompop, Zhitao Li, Xiaozhi Cao, Congyu Liao

**Affiliations:** 1 Department of Physics and Astronomy, University of California, Irvine, CA 92697, USA; lsgong1@uci.edu; 2 Department of Radiology, Stanford University, Stanford, CA 94305, USA; zihanzhj@stanford.edu (Z.Z.); kawins@stanford.edu (K.S.); 3 Department of Electrical Engineering, Stanford University, Stanford, CA 94305, USA; 4 MR Research Collaborations, Siemens Healthineers Ltd., Shanghai 200131, China; qing.li@siemens-healthineers.com; 5 Department of Radiology and Biomedical Imaging, University of California, San Francisco, CA 94143, USA; yurui.qian@ucsf.edu (Y.Q.); yang.yang4@ucsf.edu (Y.Y.); cyliao@stanford.edu (C.L.); 6 Department of Radiology, Northwestern University, Chicago, IL 60208, USA; zhitaoli@arizona.edu; 7 Room 301, Packard Electrical Engineering Building, 350 Jane Stanford Way, Stanford, CA 94305, USA

**Keywords:** radial MRI, quantitative MRI, low-field MRI, sampling trajectory

## Abstract

We present a stretched radial trajectory design that enhances image resolution in MRI by expanding k-space coverage without increasing readout duration or scan time. The method dynamically modulates gradient amplitudes as a function of projection angle, achieving square k-space coverage in 2D and cubic coverage in 3D imaging. Validation was conducted using phantom and in vivo experiments on GE and Siemens scanners at 0.55 T and 3 T. Point spread function analysis and reconstructed images demonstrated improved sharpness and clearer visualization of fine structures, including small phantom details and brain vasculature. The approach also increased T_1_ and T_2_ mapping accuracy in MRF acquisitions. The proposed strategy requires no additional scan time or gradient hardware capability, making it well-suited for MRI systems with moderate performance. It offers a simple and generalizable means to improve spatial resolution in both structural and quantitative imaging applications.

## 1. Introduction

MRI can produce high-quality images with excellent soft tissue contrast, but long acquisition times remain a major limitation. Prolonged scans can cause patient discomfort, motion artifacts, and reduced throughput, particularly in high-resolution imaging. Because MRI images are reconstructed from data acquired in the spatial frequency domain (k-space), the efficiency of k-space sampling plays a critical role in determining both image quality and scan duration. Consequently, the design of efficient k-space sampling trajectories is a key factor in enabling high-resolution imaging within clinically feasible time constraints.

Among these k-space sampling schemes, radial acquisition [1] has become a widely adopted alternative to conventional Cartesian sampling due to its unique advantages. In radial sampling, each spoke rotates around the center of k-space, inherently oversampling the low-frequency region that governs image contrast and global structure. This redundancy improves robustness against motion artifacts [2], which is particularly beneficial in dynamic imaging applications such as cardiac MRI [3]. Moreover, for half-spoke radial trajectories, since the readout begins at the k-space center rather than the periphery, this approach is well suited for ultrashort echo time (UTE) pulse sequences used to image tissues with very short T_2_ or T_2_* relaxation times, such as bone [4,5], cartilage, and myelin [6].

However, this approach suffers from a reduced effective field of view (FOV) because the outer k-space is insufficiently sampled. Since these regions encode high-frequency spatial details, inadequate sampling leads to blurred edges and loss of spatial resolution compared to Cartesian sampling, where the k-space corners are fully covered. High spatial resolution is critical in applications such as neuroimaging [7], musculoskeletal imaging [8], and quantitative techniques like magnetic resonance fingerprinting (MRF) [9,10], where tissue contrast and edge sharpness directly affect diagnostic accuracy. When using radial sampling, it is common to increase the nominal resolution by extending sampling into the outer k-space region, which is essential for resolving fine anatomical details.

In this work, we introduce a novel radial k-space sampling scheme termed “stretched radial sampling”, which dynamically increases the gradient amplitude during radial acquisition to extend coverage into the corners of k-space without increasing the number of samples per spoke, scan time, or hardware demands. This design achieves near-square k-space coverage without exceeding gradient hardware constraints or prolonging acquisition time. Both 2D and 3D implementations are validated, demonstrating that stretched radial sampling yields sharper images compared to conventional radial trajectories, without incurring additional scan time.

This article is a revised and expanded version of a conference abstract entitled “Stretched Radial Trajectory Design for Improved K-Space Coverage and Effective Image Resolution”, which was presented at the ISMRM Annual Meeting, Honolulu, Hawaii, 14 May 2025 [11].

## 2. Theory

In conventional 2D radial MRI, the sampling trajectory is generated by continuously rotating the encoding gradients around the center of k-space, in contrast to the fixed orthogonal gradients used in Cartesian sampling. For each radial projection at an angle φ (where 0≤φ<2π), the gradient waveforms are defined as a rotation of the initial readout gradient waveform along x-axis, $g_{x0}$. The gradient components, defined as g_x_ and g_y_, are given by:

(1) $$g_{x}=\cos(\varphi )g_{x0}\;g_{y}=-\sin(\varphi )g_{x0}$$

This sampling pattern results in a circular k-space coverage. Consequently, when compared to the rectangular k-space coverage of Cartesian sampling, the diagonal corners in k-space were unsampled using radial trajectories. This leads to a reduced effective resolution for a given FOV, which is a fundamental characteristic of radial acquisitions. In conventional 2D radial MRI, the maximum gradient amplitude for any projection is constrained by the physical limits of each individual gradient axis. Consequently, for any projection angle φ≠0, 0.5π, π, 1.5π, neither gradient axis operates at its maximum amplitude. To address this limitation, we propose a sampling strategy that dynamically adjusts the gradient amplitude as a function of the rotation angle φ, enabling full utilization of the available gradient strength along both axes. This modification leads to the following revised formulation:

(2) $$g_{x}=\frac{\cos(\varphi )g_{x0}}{\max\left(\left|\cos(\varphi )\right|,\left|\sin\left(\varphi \right)\right|\right)}\;g_{y}=\frac{-\sin(\varphi )g_{x0}}{\max\left(\left|\cos(\varphi )\right|,\left|\sin\left(\varphi \right)\right|\right)}$$

**Figure 1:**
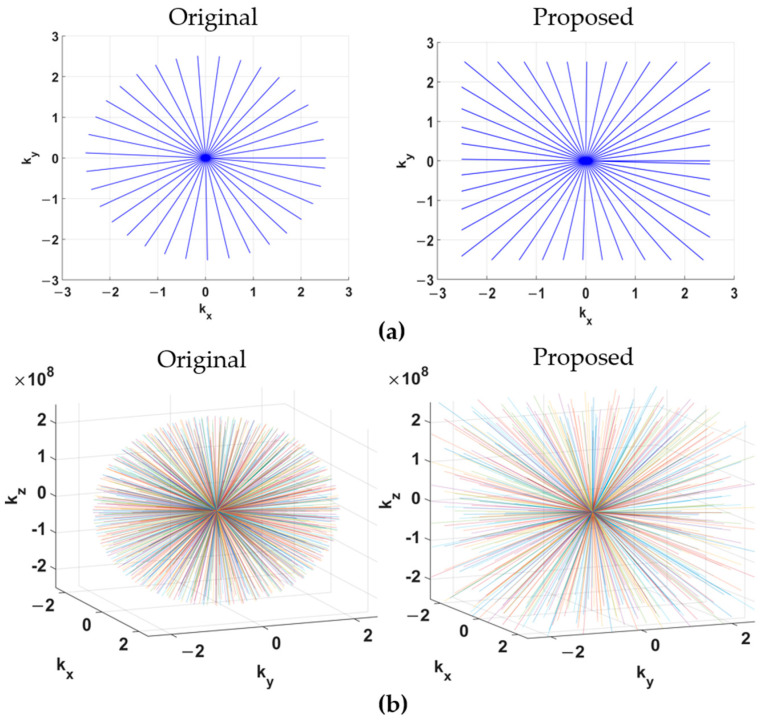
(**a**) Original and proposed 2D radial sampling trajectories; (**b**) Original and proposed 3D koosh-ball radial sampling trajectories.

The scaling term max(|cos(φ)|, |sin(φ)|) ensures that the resultant gradient vector remains within hardware constraints by rescaling its components. For each angle φ, this factor drives the dominant gradient axis to the maximum allowable amplitude while proportionally scaling the orthogonal component. This approach preserves the desired k-space trajectory while enabling a square-like coverage area akin to Cartesian sampling. It maximizes the k-space extent along the diagonals and maintains a constant readout duration without exceeding system limits on gradient amplitude or slew rate, as shown in Figure 1a.

This method generalizes naturally to 3D radial sampling patterns such as the koosh-ball trajectory, where each spoke direction is defined by two spherical coordinates: the azimuthal angle (φ), describing rotation around the k_z_ axis within the k_x_ and k_y_ plane, and the polar angle (θ), describing the inclination of the spoke relative to the k_z_ axis. A conventional 3D koosh-ball trajectory is expressed as:

(3) $$g_{x}=\cos(\varphi )g_{x0}\;g_{y}=-\sin(\varphi ){cos(\theta )g}_{x0}\;g_{z}=\sin(\varphi ){sin(\theta )g}_{x0}$$

where g_z_ is the readout gradient waveform along *z*-axis.

Applying the same dynamic scaling principle to 3D, the modified stretched-radial trajectory becomes:

(4) $$g_{x}=\frac{\cos(\varphi )g_{x0}}{\max\left(\left|\cos(\varphi )\right|,\left|\sin\left(\varphi \right)\right|\right)}\;g_{y}=\frac{-\sin(\varphi ){cos(\theta )g}_{x0}}{\max\left(abs\left(\cos(\varphi \right)\right),\left(abs\left(\sin\left(\varphi \right)\right)\right)\cdot \max\left(\left|\cos(\theta )\right|\right),\left(\left|\sin\left(\theta \right)\right|\right))}\;g_{z}=\frac{\sin(\varphi ){sin(\theta )g}_{x0}}{\max\left(\left|\cos(\varphi )\right|\right),\left(\left|\sin\left(\varphi \right)\right|\right))\cdot \max\left(\left|\cos(\theta )\right|\right),\left(\left|\sin\left(\theta \right)\right|\right))}$$

As shown in Figure 1b, this proposed trajectory covers a cubic k-space volume, analogous to 3D Cartesian sampling, in contrast to the spherical coverage of the conventional trajectory. Critically, the maximum gradient amplitude and slew rate required per physical axis for this “stretched” scheme are identical to those of the conventional scheme.

## 3. Methods

Both phantom and in vivo experiments were performed to validate the proposed method. In vivo studies were conducted with Institutional Review Board (IRB) approval, and informed consent was obtained from three healthy volunteers. To evaluate its robustness, datasets were acquired using the proposed 2D radial, 3D radial, and 3D golden-angle–ordered [12] sampling schemes and were compared with the conventional radial sampling approach. Point spread functions (PSFs) [13] were computed to assess and compare image resolution across the original and proposed 2D, 3D, and golden-angle radial trajectories.

Sampling density along representative spokes was evaluated to verify Nyquist compliance. Specifically, the maximum Δk values of the conventional and stretched trajectories along the diagonal direction were 0.0013 and 0.0022, respectively—both well below the 1/FOV threshold of 0.0045 (Supplementary Figure S1).

**Figure 2:**
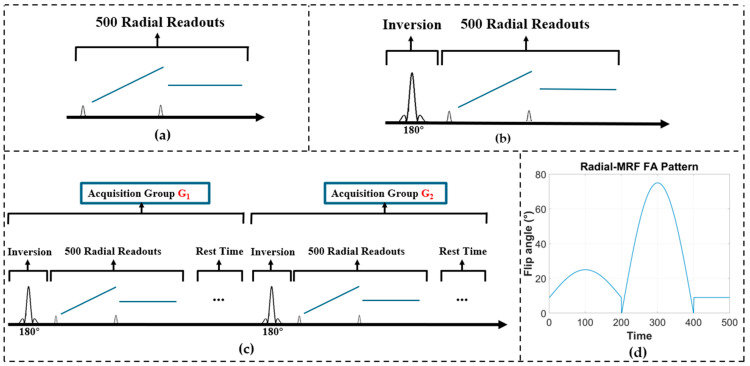
Sequence diagram of (**a**) 2D radial trajectory, (**b**) 3D MPRAGE, (**c**) two exemplary acquisition group for 3D radial-MRF, and its (**d**) FA pattern.

For contrast imaging, a half-radial GRE (gradient echo) pulse sequence was implemented with the proposed method. Both phantom and in vivo data were collected using identical parameters (TR/TE = 10/1.5 ms, FOV = 220 × 220 mm^2^, image resolution = 1 × 1 mm^2^, slice thickness = 5 mm, flip angle (FA) = 15°), as shown in Figure 2a, with reference data acquired using a conventional radial sequence for comparison. Similarly, a radial Magnetization-Prepared Rapid Gradient-Echo (MPRAGE) sequence was acquired on a Siemens 0.55T Free. Max scanner using the same protocol described above but with an adiabatic inversion pulse (Figure 2b). In addition to brain imaging, radial MPRAGE scans were also performed for the joint and liver on the 0.55T scanner using the same protocol. The acquisition speed in this scanner was primarily limited by the scanner’s moderate gradient system performance (with a maximum gradient amplitude of 25 mT/m and a maximum slew rate of 40 T/m/s) [14,15,16], making it a good application scenario for the proposed method.

For quantitative imaging, radial-MRF sequences were implemented using the proposed 3D stretched-radial sampling scheme with a non-stationary gradient-echo sequence, where each radial spoke was associated with time-varying FAs, TRs, and TEs (Figure 2c). For one MRF acquisition, a total of 500 time points were acquired, ranging from 10° to 90° across TRs, as shown in Figure 2d; while TR and TE were set to 12 ms and 0.7 ms, respectively. The acquisition was organized into 48 groups, each consisting of randomized FA and TR patterns. An identical radial-MRF sequence without the proposed enhancements was also acquired for controlled comparison. Image reconstruction was performed using a low-rank subspace approach with locally low-rank (LLR) regularization [17,18]. Notably, due to the short readout duration of radial sampling (<2 ms), image distortion and blurring caused by B_0_ inhomogeneity were significantly reduced compared with spiral readout (typically 5~10 ms in spiral MRF), making B_0_ correction [19,20] less critical in this scenario.

All experiments were performed across multiple scanner platforms and field strengths to demonstrate broad applicability, including a GE 3T UHP scanner (GE HealthCare, Chicago, IL, USA), a Siemens MAGNETOM Vida 3T scanner (Siemens Healthineers, Erlangen, Germany), and a Siemens 0.55T MAGNETOM Free.Max scanner (Siemens Healthineers, Erlangen, Germany). The 0.55 T system operated with a maximum gradient amplitude of 25 mT/m and a maximum slew rate of 40 T/m/s, while the 3 T platforms (GE and Siemens) used identical gradient waveforms with a maximum amplitude of 80 mT/m and a maximum slew rate of 100 T/m/s. Scans were conducted using vendor-specific 48-channel head coils on the GE 3T and 64-channel head/neck coils on the Siemens 3T platforms, while a 12-channel head coil was used on the Siemens 0.55T scanner. All image reconstructions were performed offline using MATLAB R2019a (The MathWorks, Inc., Natick, MA, USA) and Python 3.8 on a Linux server equipped with an Intel Xeon i7 32-core 2.8 GHz CPU, 1 TB of RAM, and an NVIDIA A6000 GPU.

Reconstructions utilized subspace + LLR for MRF and standard gridding for structural images. The reconstruction parameters for MRF used in this work were regularization weights of 5 × 10^−5^ for LLR, five subspace bases, and 40 iterations. Coil sensitivity maps were estimated using the ESPIRiT method [21] from central k-space data combined across TRs and groups. The MRF dictionary was pre-calculated using the extended phase graph method [22] with 160 T_1_ entries ([20:20:3000, 3200:200:5000] ms) and 176 T_2_ entries ([10:2:200, 220:20:1000, 1050:50:2000, 2100:100:4000] ms). The non-uniform FFT was implemented in SigPy [23].

## 4. Results

**Figure 3:**
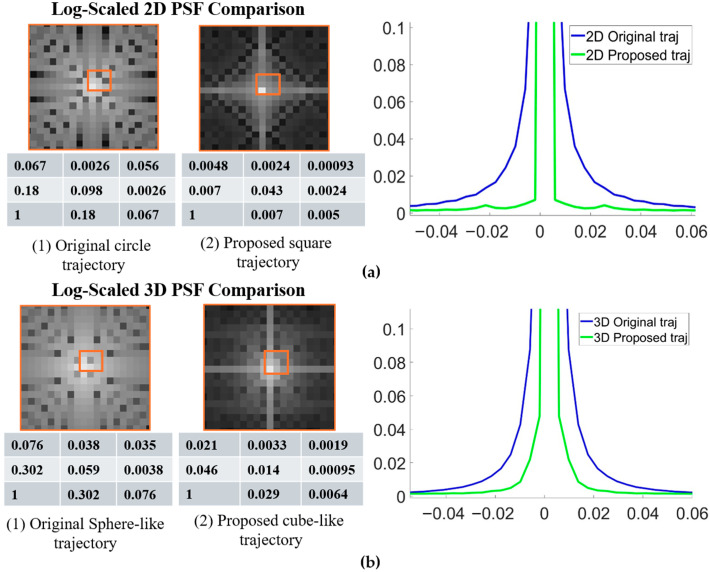
Comparison of PSFs between original and proposed radial trajectories with 1D plots and 9-point zoom-in data for (**a**) 2D radial and (**b**) 3D koosh-ball trajectories.

Figure 3a,b show comparisons of the PSFs for the original and proposed 2D and 3D radial trajectories. The proposed methods exhibit a sharper PSF profile. The signal intensity at the central region (nine points) was also reported, showing that the proposed method has higher central intensity. Quantitative analysis confirmed these observations; measurements of the full width at half maximum (FWHM) of the main lobe indicated a reduction of 10% for the 2D case, 16% for the 3D case, and 16.9% for the 3D golden-angle acquisition.

**Figure 4:**
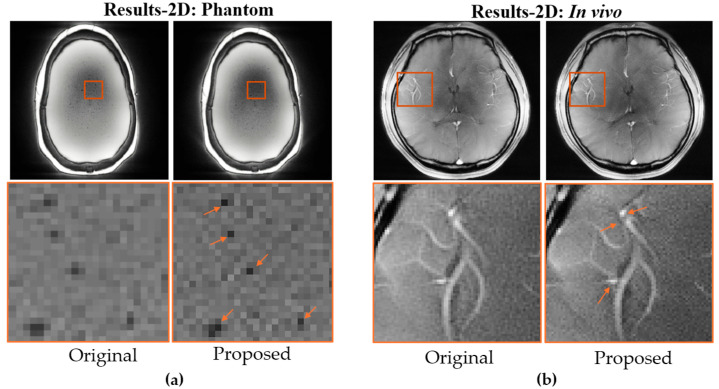
Comparison of reconstructed images using original and proposed 2D radial trajectories in (**a**) phantom and (**b**) in vivo. Zoomed-in regions and Arrows highlight improved resolution.

The improvements achieved with the proposed method were further verified through 2D phantom and in vivo GRE experiments, with results shown in Figure 4. As indicated by the arrows in Figure 4a, smaller internal imperfections within the phantom show sharper edges compared with the conventional radial acquisition. This improvement is also evident in the in vivo brain data, where the brain vasculature is visualized with greater clarity, as highlighted in the zoomed-in regions of Figure 4b.

**Figure 5:**
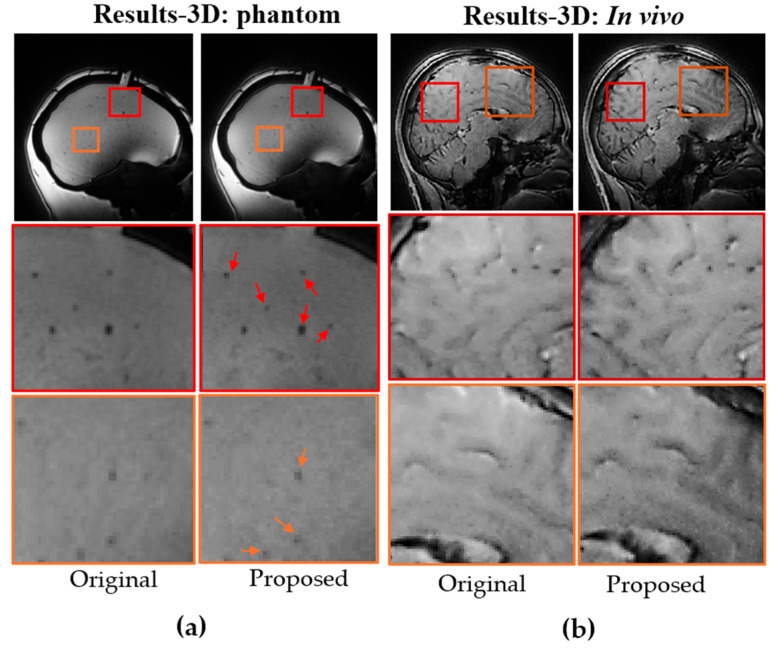
Comparison of reconstructed images using original and proposed 2D radial trajectories in (**a**) phantom and (**b**) in vivo. Zoomed-in regions and arrows highlight improved resolution.

**Figure 6:**
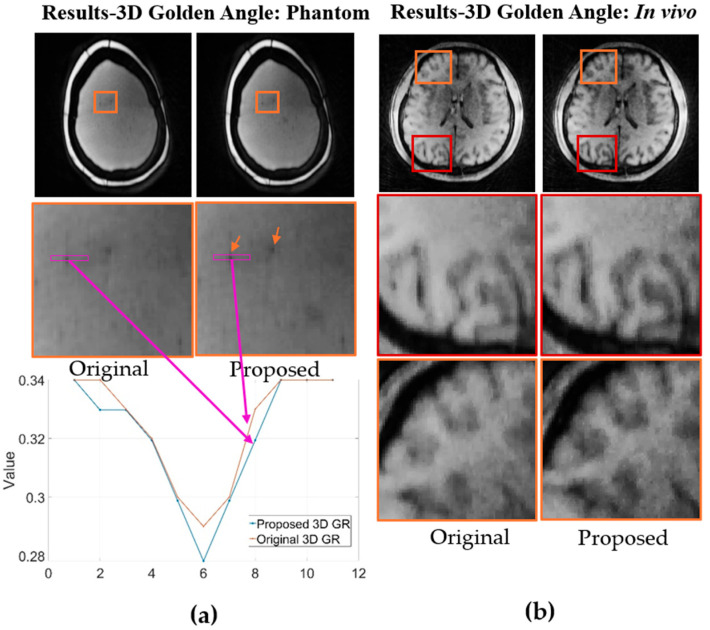
Comparison of reconstructed images using original and proposed 3D Golden-angle trajectories in (**a**) phantom and (**b**) in vivo. Line plots in (**a**) show signal profiles at indicated positions. Zoomed-in regions and arrows highlight improved resolution.

The benefits of the proposed method were further substantiated in 3D imaging using the same protocol as the 2D GRE sequence, but with 3D trajectories. The improvements were even more substantial, consistent with the PSF simulations. As shown in Figure 5a and b, both phantom and in vivo brain images exhibit higher resolution and better-defined edges, as indicated by the red arrows. The enhancement in edge definition was further verified with golden-angle view ordering (Figure 6a,b). A line profile across a small bubble in the phantom (Figure 6a) shows increased sharpness at the bubble’s edge. Comparable enhancements are visible in the in vivo brain images within the zoomed-in regions of Figure 6b. In addition to improved resolution and feature definition, the proposed 3D method with golden-angle ordering also provided superior tissue contrast compared to the conventional 3D radial approach.

**Figure 7:**
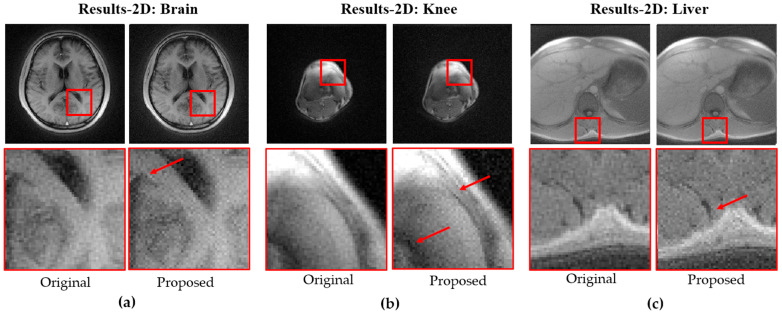
Comparisons of 3D MPRAGE images using original and proposed trajectories for (**a**) brain, (**b**) knee, and (**c**) liver, with zoomed views showing differences (red arrows).

Figure 7 shows 3D MPRAGE images acquired on a Siemens 0.55T Free.Max scanner using the original and proposed trajectories for the brain (a), knee (b), and liver (c), with zoomed-in views highlighting differences as indicated by the red arrows.

In vivo quantitative evaluation demonstrated consistent improvements in edge sharpness, quantified using the 10–90% edge-rise distance method [24], where the inverse rise distance was computed from local edge profiles and summarized as the median value across the image. On the 3 T system, the 2D and 3D brain scans showed median edge-sharpness increases of 1.8% and 2.6%, respectively. On the 0.55 T system, the 2D brain exhibited a 3.2% increase, while ROI-based analyses of the knee and liver showed gains of 1.1% and 0.3%, respectively. These results confirm that the proposed stretched trajectory improves effective resolution across different vendors of different field strength.

**Figure 8:**
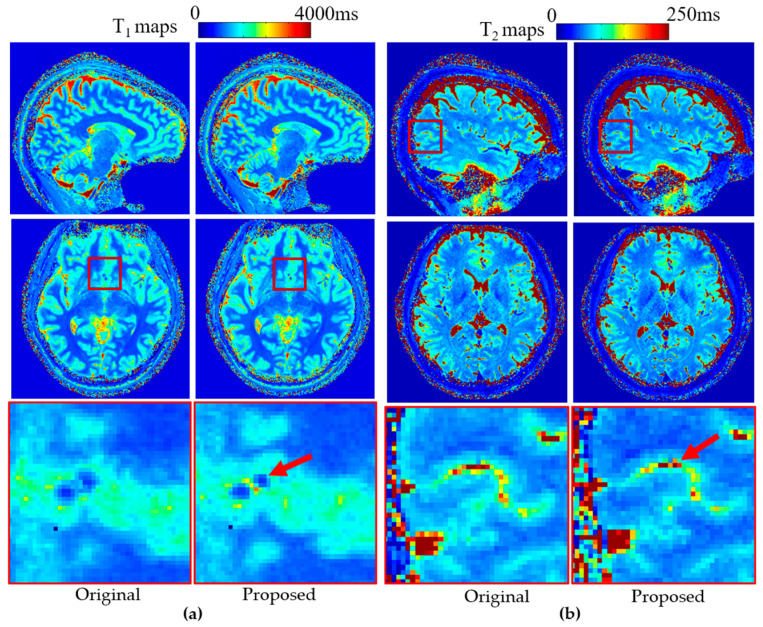
Quantitative (**a**) T_1_ and (**b**) T_2_ maps obtained using 3D radial magnetic resonance fingerprinting (MRF) from data acquired with the original and proposed trajectories. With zoomed views showing differences (red arrows).

To demonstrate the broad applicability of the proposed method, the stretched radial trajectory was implemented within a 3D radial MRF pulse sequence. Representative results from a brain scan are shown in Figure 8. Quantitative T_1_ (Figure 8a) and T_2_ (Figure 8b) maps acquired using the proposed stretched radial trajectory exhibit improved edge sharpness compared with those obtained using the conventional radial trajectory. These enhancements, visible in the zoomed-in regions, illustrate the method’s effectiveness in preserving fine structural detail and highlight its potential for improving quantitative imaging. B_0_ effects were further evaluated by reconstructing MRF data with and without B_0_ correction (Supplementary Figure S2), showing negligible differences and confirming minimal off-resonance sensitivity due to the short (<2 ms) radial readout [25].

## 5. Discussion

In this study, we introduced a stretched radial trajectory that significantly improves k-space coverage and effective image resolution without increasing scan time or exceeding gradient hardware limits. By dynamically modulating gradient amplitudes across projection angles, the proposed method enables square-like (2D) and cube-like (3D) sampling geometries while maintaining compatibility with conventional hardware. Compared with conventional radial sampling, which typically requires extending the spoke length to match the nominal resolution of Cartesian sampling, the proposed trajectory achieves broader k-space coverage and reaches a larger maximum spatial frequency (k_max_) within the same acquisition window. PSF analysis confirmed these improvements, showing a narrower main lobe (lower FWHM) and suppressed sidelobes along the diagonal direction, corresponding to higher effective resolution. A slight increase in orthogonal sidelobe intensity was observed, which will be considered in future trajectory optimization to further improve PSF uniformity.

The expanded coverage enhances the encoding of high-frequency components, particularly in the diagonal directions that are typically underrepresented in circular radial acquisitions. Importantly, this improvement is achieved without increasing the number of samples per spoke, the scan time, or the hardware demands. As a result, the proposed method improves sampling efficiency and is fully compatible with existing gradient systems and reconstruction frameworks [26]. These advantages make the proposed approach particularly suitable for scenarios requiring high resolution but limited by scan time or hardware performance, such as low-field, portable, or time-constrained MRI applications. For example, achieving high-resolution imaging in low-performance MRI systems with restricted gradient amplitude is often challenging. The 0.55T scanner used in this study, for instance, has a maximum gradient strength of only 25 mT/m and a maximum slew rate of 40 T/m/s, which limits its ability to fast encode the k-space. In such cases, the proposed method is particularly effective, as it fully utilizes all three gradient axes (G_x_, G_y_, G_z_) to expand k-space coverage. This results in improved effective resolution without increasing scan time or requiring additional hardware resources.

Gradient delay correction was implemented where gradient delay estimation was based on detecting k-space zero-crossing points [27]. Although eddy current effects were not explicitly modeled in this study, their influence is expected to be limited due to the short readout duration. Future work will include quantitative characterization and compensation of these effects.

One potential drawback of this approach is the risk of increased peripheral nerve stimulation (PNS), due to the more effective simultaneous use of the G_x_, G_y_, and G_z_ gradients. However, this concern is mitigated in lower-performance MRI systems, where the maximum slew rate remains well below PNS safety thresholds. For example, the 0.55T scanner used in this study has a maximum slew rate of 40 T/m/s, which is well below the level typically associated with PNS induction. This approach also poses minimal risk in head-only gradient systems, where gradient switching is confined to the cranial region and the volume of tissue exposed to time-varying magnetic fields is smaller. Prior studies have shown that dedicated head gradient coils exhibit substantially higher PNS thresholds than body gradients. For instance, an insertable head coil demonstrated thresholds of approximately 108 mT/m and 156 T/m/s on its most sensitive axis [28], well above typical imaging demands. Moreover, modeling and experimental analyses indicate that head-only gradient systems operate with a wider safety margin relative to IEC and FDA PNS guidelines [29]. In addition, we evaluated the gradient waveforms using the Siemens sequence simulator. The mean of ∫Gx^2^ dt across the entire sequence was 28.5 (mT/m)^2^ for the original design and 33.1 (mT/m)^2^ for the stretched trajectory, indicating a 16.1% higher average gradient-squared level. This modest increase indicates slightly greater simultaneous gradient activity but remains within accepted safety margins for standard and head-only systems.

Another potential limitation of the proposed approach is undersampling along the radial direction, which could lead to image artifacts or reduced signal fidelity. However, this issue can be effectively mitigated by increasing the sampling rate, which is straightforward to implement in MRI pulse sequences. In practice, these adjustments ensure adequate sampling density without requiring longer scan times or additional hardware modifications.

While advanced designs such as multi-axis spiral, cone [30], or stack-of-spiral trajectories [31] can also enhance diagonal or volumetric k-space coverage, they belong to fundamentally different trajectory families that involve distinct gradient encoding schemes and timing structures, and therefore cannot be directly compared within the same reconstruction framework. The proposed stretched-radial approach instead represents a minimal-modification extension of conventional radial and SPI-based acquisitions, improving diagonal coverage and effective resolution without additional calibration or sequence-level reimplementation. Furthermore, the same principle can be applied to cylindrical or sphere-like 3D k-space coverage, providing improved sampling uniformity while remaining fully compatible with existing pulse sequence and reconstruction pipelines.

In addition, reconstruction efficiency was evaluated. The 3D reconstruction required approximately 200 s with an input memory load of ~480 MB, whereas the 2D reconstruction was completed in ~10 s using ~35 MB of input memory. The 3D radial MRF reconstruction required an input memory load of ~1.5 GB and a total computation time of ~25 min.

In this work, the proposed method was validated across 2D and 3D radial trajectories, including those with golden-angle view ordering, and was further evaluated in MRF for high-resolution quantitative T_1_ and T_2_ mapping. Beyond radial trajectories, the same principle can be extended to other non-Cartesian sampling strategies that rely on trajectory rotation during acquisition, such as 3D spiral projection imaging [32], cones, and hybrid 3D radial-EPI [33]. In future work, we will explore the potential of applying the stretched-trajectory design to 3D UTE-MRF [34] for quantifying tissues with extremely short T_2_ and T_2_* values, such as cartilage and myelin, and for investigating its applicability in dynamic imaging scenarios, including cardiac imaging.

## 6. Conclusions

The stretched radial trajectory provides a simple and effective modification to conventional radial MRI, enabling improved spatial resolution without increasing scan time or hardware demand. Its compatibility with 2D, 3D, golden-angle, and MRF acquisitions was validated across multiple scanners and field strengths, demonstrating enhanced visualization of fine anatomical structures and improved quantitative mapping. Owing to its simplicity, generalizability, and hardware efficiency, the stretched radial trajectory offers a practical solution for improving image quality, particularly in low-performance MRI systems.

## Data Availability

The 2D and 3D stretched radial sequences and raw k-space datasets are available upon request.

## Supplementary Materials

The following supporting information can be downloaded at: https://www.mdpi.com/article/10.3390/bioengineering12111152/s1, Figure S1: Comparison of k-space sampling density between the conventional and stretched radial trajectories. Δk spacing was measured along representative spokes, including the diagonal direction where spacing is maximal. The calculated Nyquist threshold (1/FOV = 0.0045, normalized k units) is indicated by the dashed line. The maximum Δk values were 0.0013 for the conventional and 0.0022 for the stretched trajectory, both remaining below the Nyquist limit, confirming sufficient sampling density across k-space; Figure S2: Evaluation of B_0_ inhomogeneity effects in 3D radial MRF reconstructions. Quantitative T_1_ maps were reconstructed with and without B_0_ correction for both the conventional and stretched trajectories. The negligible differences between the two reconstructions confirm that the short radial readout duration (<2 ms) effectively minimizes off-resonance sensitivity in both designs.
