# Supplementary material for: Stretched Radial Trajectory Design for Efficient MRI with Enhanced K-Space Coverage and Image Resolution
Source: Bioengineering (Basel). 2025 Oct 24;12(11):1152. doi: 10.3390/bioengineering12111152 (PMC12649564; doi:10.3390/bioengineering12111152)
Supplement: Supplementary file 1 [file bioengineering-12-01152-s001.zip › bioengineering-3917341-supplementary.pdf]

Supplementary materials for Stretched Radial Trajectory Design for Efficient MRI with Enhanced K-space Coverage and Image Resolution

Li Song Gong[1,2], Zihan Zhou[2,3], Qing Li[4], Yurui Qian[5], Yang Yang[5], Kawin Setsompop[2,3], Zhitao Li[6], Xiaozhi Cao[2,3*], Congyu Liao[5]

Introduction

The supplementary materials provide additional data and analyses supporting the findings presented in the main manuscript *"Stretched Radial Trajectory Design for Efficient MRI with Enhanced K-space Coverage and Image Resolution."* These materials include quantitative validation figures and supporting datasets used to verify sampling density, PSF characteristics, and off-resonance sensitivity.

Supplementary Figure. S1

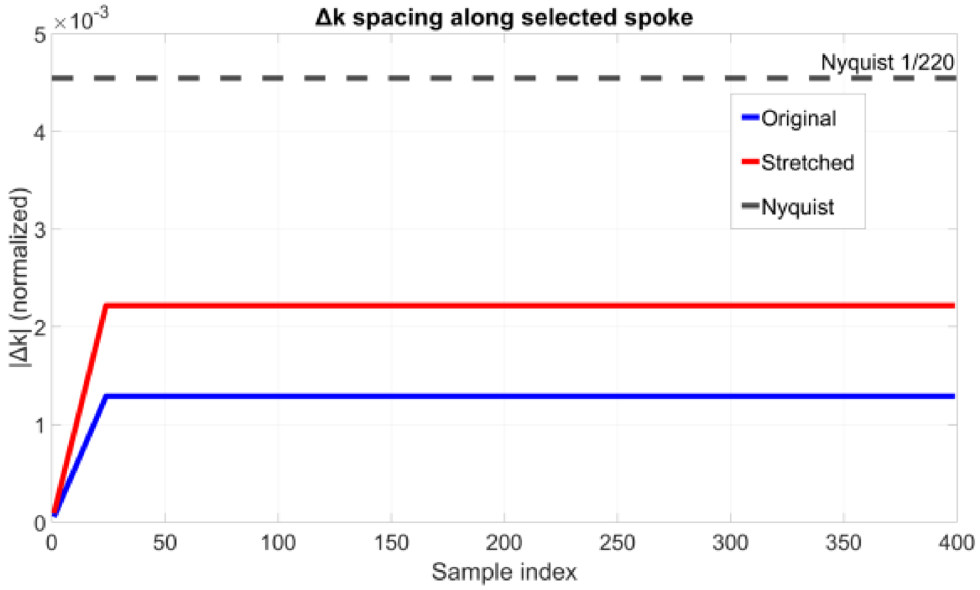

Comparison of k-space sampling density between the conventional and stretched radial trajectories. Δk spacing was measured along representative spokes, including the diagonal direction where spacing is maximal. The calculated Nyquist threshold (1/FOV = 0.0045, normalized k units) is indicated by the dashed line. The maximum Δk values were 0.0013 for the conventional and 0.0022 for the stretched trajectory, both remaining below the Nyquist limit, confirming sufficient sampling density across k-space.

Supplementary Figure. S2

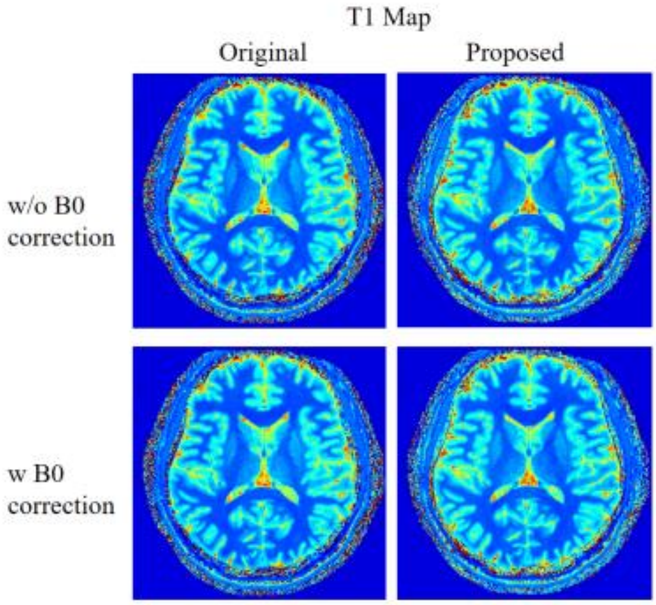

Evaluation of B_0 inhomogeneity effects in 3D radial MRF reconstructions. Quantitative T_1 maps were reconstructed with and without B_0 correction for both the conventional and stretched trajectories. The negligible differences between the two reconstructions confirm that the short radial readout duration (< 2 ms) effectively minimizes off-resonance sensitivity in both designs.
